# Ability of Heart Rate Recovery and Gait Kinetics in a Single Wearable to Predict Frailty: Quasiexperimental Pilot Study

**DOI:** 10.2196/58110

**Published:** 2024-10-03

**Authors:** Reshma Aziz Merchant, Bernard Loke, Yiong Huak Chan

**Affiliations:** 1 Division of Geriatric Medicine Department of Medicine National University Hospital Singapore Singapore; 2 Biologic Technik Singapore Singapore; 3 Biostatistics Unit Yong Loo Lin School of Medicine National University of Singapore Singapore Singapore

**Keywords:** falls, fall prevention, wearables, older adult, community dwelling older adults, gait, gait kinetics, gait analysis, biomechanics, sensors, gerontology

## Abstract

**Background:**

Aging is a risk factor for falls, frailty, and disability. The utility of wearables to screen for physical performance and frailty at the population level is an emerging research area. To date, there is a limited number of devices that can measure frailty and physical performance simultaneously.

**Objective:**

The aim of this study is to evaluate the accuracy and validity of a continuous digital monitoring wearable device incorporating gait mechanics and heart rate recovery measurements for detecting frailty, poor physical performance, and falls risk in older adults at risk of falls.

**Methods:**

This is a substudy of 156 community-dwelling older adults ≥60 years old with falls or near falls in the past 12 months who were recruited for a fall prevention intervention study. Of the original participants, 22 participants agreed to wear wearables on their ankles. An interview questionnaire involving demographics, cognition, frailty (FRAIL), and physical function questions as well as the Falls Risk for Older People in the Community (FROP-Com) was administered. Physical performance comprised gait speed, timed up and go (TUG), and the Short Physical Performance Battery (SPPB) test. A gait analyzer was used to measure gait mechanics and steps (FRAIL-functional: fatigue, resistance, and aerobic), and a heart rate analyzer was used to measure heart rate recovery (FRAIL-nonfunctional: weight loss and chronic illness).

**Results:**

The participants’ mean age was 74.6 years. Of the 22 participants, 9 (41%) were robust, 10 (46%) were prefrail, and 3 (14%) were frail. In addition, 8 of 22 (36%) had at least one fall in the past year. Participants had a mean gait speed of 0.8 m/s, a mean SPPB score of 8.9, and mean TUG time of 13.8 seconds. The sensitivity, specificity, and area under the curve (AUC) for the gait analyzer against the functional domains were 1.00, 0.84, and 0.92, respectively, for SPPB (balance and gait); 0.38, 0.89, and 0.64, respectively, for FRAIL-functional; 0.45, 0.91, and 0.68, respectively, for FROP-Com; 0.60, 1.00, and 0.80, respectively, for gait speed; and 1.00, 0.94, and 0.97, respectively, for TUG. The heart rate analyzer demonstrated superior validity for the nonfunctional components of frailty, with a sensitivity of 1.00, specificity of 0.73, and AUC of 0.83.

**Conclusions:**

Agreement between the gait and heart rate analyzers and the functional components of the FRAIL scale, gait speed, and FROP-Com was significant. In addition, there was significant agreement between the heart rate analyzer and the nonfunctional components of the FRAIL scale. The gait and heart rate analyzers could be used in a screening test for frailty and falls in community-dwelling older adults but require further improvement and validation at the population level.

## Introduction

The number of older adults aged ≥65 years is projected to double to 1.5 billion by 2050 [1]. Populations in Asia Pacific countries, such as Singapore and Taiwan, are aging the fastest. Aging is a risk factor for chronic diseases, falls, dementia, frailty, and disability. Countries with a rapidly aging population will face escalating health and social care costs. For instance, in Hungary, the health care cost for an 80-year-old man was 15.8 times higher than that for a 20-year-old man in 2015 [2]. The World Health Organization (WHO) defines healthy aging as the process of developing and maintaining functional ability, which includes both physical and cognitive function and enables well-being [3]. Prevention of frailty, falls, and dementia is a public health priority in many countries worldwide [2,4]. As a result, there is a rise in the following 2 parallel trends: (1) development of digital technologies for continuous population-level assessment and monitoring and (2) national programs for healthy longevity that take a life-course approach [2].

Frailty is a dynamic state of reduced physiological reserve that predisposes older adults to adverse events when exposed to stressors [5]. It is reversible before the onset of disability through multidimensional interventions such as exercise, social activities, protein intake, and a nutrient-enriched diet [6,7]. The prevalence of frailty in community-dwelling older adults varies between 7% and 46.3% depending on the frailty screening tools used and population studied [8-10]. There are many diagnostic criteria for frailty such as the frailty phenotype described by Fried et al [11], which consists of 5 criteria: unintentional weight loss, self-reported exhaustion, weakness, slow walking speed, and low levels of physical activity. The FRAIL scale is a questionnaire-based screening tool that captures fatigue, climbing 1 flight of stairs (resistance), walking 50 meters (aerobic), unintentional weight loss, and ≥5 chronic illnesses [12]. Besides age, frailty is a well-recognized risk factor for falls, but to date, there are no gold standard validated tools for frailty and falls assessment [5,13,14]. In addition, current tools have limitations such as recall bias, which may limit their use with older adults with cognitive impairment; being resource intensive; and requiring space, equipment, and trained professionals to conduct the assessments [12]. Therefore, other sources of information such as that from sensor-based instruments can provide an additional quantified data source for frailty assessment including gait speed, physical activity, and heart rate recovery (HRR) [15,16].

Of the 5 frailty phenotypic criteria, 3 are directly related to individual locomotor mobility: (1) self-reported exhaustion, (2) gait speed, and (3) physical activity. Gait speed is a diagnostic screening tool for frailty and sarcopenia and is a component of motoric cognitive risk syndrome, which is a prodrome of dementia [17-19]. The advancement of wearable technology, especially from the consumer electronics segment, is very suited to continuous remote monitoring of motion-related parameters such as physical activity and gait speed. Mueller et al [17] demonstrated that long-term digital monitoring of mobility in frail older adults was reliable and reflective of in-clinic performance. Beyond gait and physical activity, digitally recorded HRR is a new area gaining recognition in frailty research [18,20,21]. HRR, like heart rate variability, is a surrogate for autonomic function, and both are well-recognized biomarkers for frailty, fatigue, chronic inflammation, mortality, insulin resistance, and a higher risk of cardiovascular events [16,20,22-24].

One of the common reasons for adoption of wearables among older adults is continuous data collection and ease of use. Consumer electronic brands such as Apple and Samsung have smartwatches (Apple Watch and Samsung Galaxy Watch, respectively) that cover multiple domains in motion, activity, and cardiovascular health (heart rate, electrocardiogram). However, some wearable functions require specific actions by the users to trigger the measurement, which may be challenging for older adults especially those with cognitive impairment. Standardized frailty screening using sensor-based wearables will enable population-level screening in a cost-effective manner with necessary upstream interventions to delay the onset of disability. However, a recent scoping review highlighted numerous gaps in adoption such as validation of clinical efficacy and lack of data expertise among clinicians [19]. The aim of this study was to evaluate the accuracy and validity of using continuous digital monitoring wearable devices to detect frailty and poor physical performance in older adults at risk of falls.

## Methods

### Study Participants

This is a substudy of 156 community-dwelling older adults ≥60 years old with falls or near falls in the past 12 months who were recruited for a fall prevention intervention study from community and primary care centers in Singapore. Recruited participants were able to provide consent, adhere to instructions, and be ambulant. Participants from nursing homes, with a pacemaker or defibrillator, and with any underlying psychiatric conditions were excluded. In addition to participating in the fall prevention program, participants were offered the option to wear devices on their ankles for gait analysis. Only those who consented were included in this study.

### Ethical Considerations

This study conformed to the principles of the Declaration of Helsinki and was approved by The National Healthcare Group Domain Specific Review Board (Reference: 2019/00650). Written informed consent was obtained from all participants involved in the study. Data analysis was conducted in an anonymized manner, and no specific compensation was provided for participants who agreed to wear the wearables.

### Covariates

Trained research assistants administered study questionnaires on demographics, education, depression, frailty, function, physical performance, and falls. Frailty was measured using the FRAIL (fatigue, resistance, aerobic, number of illnesses, and loss of weight) questionnaire. FRAIL has a maximum score of 5: 0 is considered robust; 1 and 2 are considered prefrail; and 3, 4, and 5 are considered frail. Participants who experienced at least 1 fall in the past 12 months were considered fallers. Physical performance comprised 4-meter gait speed, the Short Physical Performance Battery (SPPB) test, and the timed up and go (TUG) test. The SPPB includes 3 components (balance, gait speed, and chair stand) with a maximum score of 12 points (4 points per component). Participants’ baseline falls risk was assessed using the Falls Risk for Older People in the Community (FROP-Com) tool developed by the National Ageing Research Institute Australia [25]. It is made up of 13 risk factors, with total scores ranging from 0 to 60: individuals scoring ≤11 points are considered to have a low falls risk, those scoring 12 to 18 points are considered to have a moderate risk, and individuals scoring ≥19 points have a high risk. These tests form the reference standard for the purpose of this study.

### Wearable Device

#### Overview

**Figure 1:**
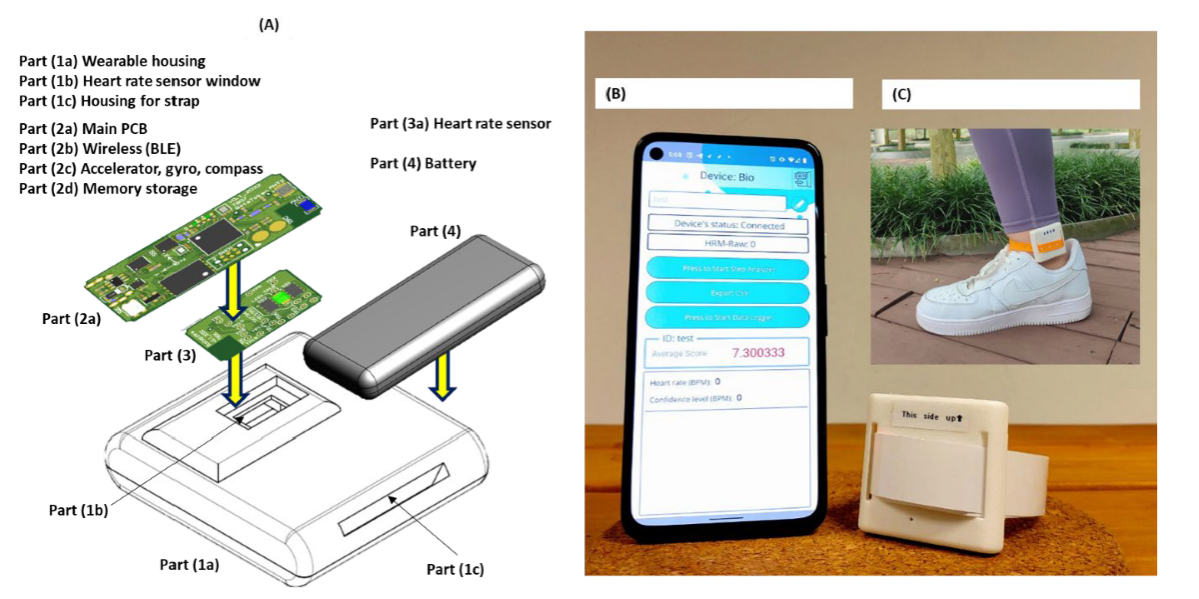
(A) The wearable device, (B) the Android smartphone app, and (C) wearing the device on the inner right ankle. BLE: Bluetooth low energy; PCB: printed circuit board.

The wearable device is shown in Figure 1. Within the waterproof housing, there are 3 main components: the main printed circuit board (PCB) with a wireless Bluetooth module, motion sensors (accelerator, gyroscope, and compass), and memory storage (Figure 1A). A second PCB has a heart rate sensor using photoplethysmography. The wearable device has a rechargeable battery and is designed to allow wireless pairing with the Android smartphone app. Thereafter, the wearable device operates 100% autonomously 24/7 for up to 3 to 4 days. The onboard battery, when fully charged, can support the various sensors and 2 PCBs. The onboard memory allows continuous data acquisition from the motion and heart rate sensors. Since the wearable device requires autonomous operation for continuous data collection, it is not feasible for either researchers or participants to manipulate any controls to maintain device operation. The 3 to 4 days of continuous operation cater for longer periods of data collection, so researchers can conduct less frequent visits with the participants.

The wearable used in this study was purpose-built to conduct research. The features and functions are not available in most off-the-shelf consumer devices. This wearable is equipped with large onboard memory (Figure 1A) to allow continuous autonomous operation for multiple days of high-resolution raw data (off-the-shelf devices do not store raw data). The post-data processing software is programmable to derive new parameters (off-the-shelf devices do not provide software programmability) as new use cases develop. Both the raw data and postprocessed data can be uploaded to any cloud or clinical database for further artificial intelligence or other analytics.

The wearable device is designed to be worn on the inner side of the right ankle (Figure 1C). The heart rate sensor window in Figure 1A must be well-placed onto the participant’s skin. Loose fitment on the skin can cause inaccurate heart rate measurements. The Android smartphone app (Figure 1B) is used to control the device and to start and stop the device data recording. After data collection is complete, the desktop device control (Figure S1 in Multimedia Appendix 1) is used to download the data onto a desktop computer. This data file is analyzed using the desktop analytics software in which there are 3 algorithms: (1) gait analyzer, (2) heart rate analyzer, and (3) daily living analyzer.

#### Gait Analyzer

The gait analyzer can process motion sensor data (including accelerometer, gyroscope, and compass data) as input for the biomechanics model. In this study, the gait analyzer’s output was validated using a gait lab equipped with a force plate and motion capture system to ensure accuracy. Cross-validation revealed an average deviation of 9% between the gait analyzer’s results and those obtained in the gait lab for key parameters such as cadence, toe-off moment, and swing power.

For this study, the gait analyzer was used to measure gait mechanics, including cadence, toe-off moment, and swing power. Additionally, it was used for step detection and step count. In the context of step counting, a continuous 40 steps was considered as a single step count.

#### Daily Living Analyzer

In many studies, the daily living activity level is categorized [26] into 3 groups: (1) light activity while sitting, (2) household tasks of a moderate effort, and (3) cycling (high effort). Using accelerometer data from the wearable device to compute energy level output by the participant is a common method in similar research studies [27]:

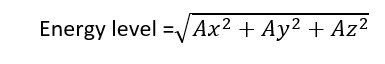



where A denotes acceleration data and x, y, z are the x, y, and z axes, respectively.

This energy level was normalized to an energy level of a walking speed (cadence) of 1 step/second. Using this as a reference, the 3 categories of daily activity were derived as (1) light activity while sitting: <10% of the energy level (no walking detected); (2) household tasks of a moderate effort: ≥10% to <30% of the energy level (intermittent walking of 1 step/second); (3) cycling moderately: ≥30% of the energy level (continuous walking of 1 step/second).

The energy level was further normalized on an hourly basis. Based on the aforementioned categorization of energy levels from the wearable device data analysis, the average total daily energy in calories was calculated. For each participant, the average total daily energy (in kilocalories) was classified as low (<521 kcal/day), middle (521-770 kcal/day), or high (>770 kcal/day).

#### Heart Rate Analyzer

A common heart rate analysis related to frailty is HRR. Although HRR takes various forms, the typical protocol for HRR [28] assessment requires the participant to (1) exercise until a predefined condition is reached, usually when achieving a percentage of the maximum heart rate based on age, and (2) take the maximum heart rate, rest for approximately 60 seconds to 120 seconds, and measure heart rate again. HRR is defined as the difference between the maximum heart rate and the heart rate after rest. A higher HRR represents better fitness in the participant.

In daily activities, the conventional protocol for assessing HRR is impractical due to the deliberate actions required by the participant. In our study, we developed an automated method to record both the maximum heart rate and recovery heart rate. Our analytics software identified instances when the energy level intensity (as defined by the daily living analyzer) exceeded 30%. It concurrently tracked this level until a peak heart rate was registered as the maximum heart rate. Subsequently, within a 5-minute interval (matching the sampling rate of the heart rate sensor data recording), the next heart rate measurement was recorded as the recovered heart rate. The difference between these 2 heart rates—the maximum heart rate and the recovered heart rate—defined HRR. The analytics software identified various episodes of this protocol and computed the average HRR as a singular measurement for each participant.

### Statistical Analysis

The data were analyzed using a diagnostic test framework to verify the predictive value of the gait analyzer and heart rate analyzer compared with the reference standards. A total of 10 experimental data sets resulted from the combination of the 5 reference standards and 2 screener tests (5 reference standards × 2 screener tests = 10 data sets).

For each experimental data set, a 2 × 2 diagnostic test was established (Figure S2 in Multimedia Appendix 1). Based on this diagnostic test, sensitivity and specificity were calculated and plotted on the receiver operating characteristic (ROC) curve. The optimal cutoff thresholds (Figure S3 in Multimedia Appendix 1) for the gait analyzer and heart rate analyzer were determined when the Youden index (max [sensitivity + specificity – 1]) was maximized. The optimal cutoff threshold occurs at the Youden index. To calculate the corresponding area under the curve (AUC), the ROC curve was approximated using a series of trapezoids. The AUC is thus the sum of all the areas of each trapezoid, calculated using the conventional trapezoid area method (see Figure S4 for the AUC calculation method).

### Classifying Functional Versus Nonfunctional Tests

Some of the reference standards, such as the SPPB and the FRAIL scale, include both functional components related to physical performance measurements and nonfunctional components (such as chronic diseases or unintentional weight loss). When analyzing the results of the SPPB, which consists of 3 separate tests, the 5x sit-to-stand (5x-STS) had a higher rate of false classification. As a result, we focused on comparing performance data from the wearable from only 2 subcomponents of the SPPB: gait and balance. For the FRAIL scale, only fatigue, walking 50 meters, and climbing 1 flight of stairs were compared.

To support the functional and nonfunctional components, the wearable data provided by the gait analyzer were classified as functional, and data from the heart rate analyzer were classified as nonfunctional data.

## Results

### Participants

The mean participant age was 74.6 years, with 5.6 years of education (Table 1). Among the 22 participants, 9 (41%) were robust, 10 (46%) were prefrail, and 3 (14%) were frail.

**Table 1:** Demographics of the study population (n=22).

Variables		Results
Age (years), mean (SD)		74.62 (7.2)
**Frailty status, n (%)**		
	Robust	9 (41)
	Prefrail	10 (45)
	Frail	3 (14)
≥1 fall in the past year, n (%)		8 (36)
≥1 ADL^a^ limitation, n (%)		4 (18)
≥1 IADL^b^ limitation, n (%)		4 (18)
Gait speed (m/s), mean (SD)		0.83 (0.30)
Total SPPB^c^ score, mean (SD)		8.86 (3.36)
**SPPB component scores, mean (SD)**		
	Gait	3.32 (0.99)
	Balance	3.00 (1.21)
	5x sit-to-stand	2.27 (1.45)
TUG^d^ (seconds), mean (SD)		13.75 (8.68)
TUG (seconds), range		3.39-75.61

^a^ADL: activity of daily living.

^b^IADL: instrumental activity of daily living.

^c^SPPB: Short Physical Performance Battery.

^d^TUG: timed up and go.

### Gait Analyzer

#### Reference Standard Versus Gait Analyzer

Table 2 shows the comparison of the gait and heart rate analyzers with the reference standard results. The reference standard test results (gait and balance in the SPPB, TUG, and gait speed) were also compared against the gait analyzer results using an ROC (Figure S3 in Multimedia Appendix 1), resulting in an AUC >0.8 (good validity).

**Table 2:** Summarized results for the gait analyzer and heart rate analyzer versus the reference standards.

Variables		Optimal cutoff	Sensitivity	Specificity	True positive rate	False positive rate	Positive predictive value	Negative predictive value	Area under the curve
**Gait analyzer results**									
	SPPB^a^ (gait, balance, and chair stand)	3	0.63	0.93	0.63	0.07	0.83	0.81	0.78
	SPPB (gait and balance only)	3	1.00	0.84	1.00	0.16	0.50	1.00	0.92
	Frail (functional and nonfunctional)	3	0.38	0.89	0.38	0.11	0.83	0.50	0.64
	Frail (functional)	3	0.45	0.91	0.45	0.09	0.83	0.63	0.68
	Frail (nonfunctional)	N/A^b^	N/A	N/A	N/A	N/A	N/A	N/A	N/A
	FROP-Com^c^	3	0.45	0.91	0.45	0.09	0.83	0.63	0.68
	Gait speed	3	0.60	1.00	0.60	0.00	1.00	0.75	0.80
	Timed up and go	3	1.00	0.94	1.00	0.06	0.83	1.00	0.97
**Heart rate analyzer results**									
	SPPB (gait, balance, and chair stand)	–14	0.50	0.75	0.50	0.25	0.50	0.75	0.63
	SPPB (gait and balance only)	–14	1.00	0.73	1.00	0.27	0.25	1.00	0.86
	Frail (functional and nonfunctional)	–14	0.57	1.00	0.57	N/A	1.00	0.63	0.79
	Frail (functional)	N/A	N/A	N/A	N/A	N/A	N/A	N/A	N/A
	Frail (nonfunctional)	–14	1.00	0.73	1.00	0.27	0.25	1.00	0.86
	FROP-Com	–15	0.75	0.75	0.75	0.25	0.60	0.86	0.75
	Gait speed	–14	0.67	0.78	0.67	0.22	0.50	0.88	0.72
	Timed up and go	–14	1.00	0.73	1.00	0.27	0.25	1.00	0.86

^a^SPPB: Short Physical Performance Battery Test.

^b^Not applicable.

^c^FROP-Com: Falls Risk for Older People in the Community.

#### SPPB Versus Gait Analyzer

The overall results for the SPPB (gait, balance, and 5x-STS) demonstrated high specificity (0.93), a positive predictive value (PPV) of 0.83, and a negative predictive value (NPV) of 0.81 (as shown in Table 2). The AUC was 0.78.

Interestingly, when focusing on the subdomains of gait and balance, the overall results showed even better performance, with higher sensitivity, true positive rate (TPR), and NPV and an AUC of 0.92 (Table 2) compared with the broader SPPB assessment that included the 5x-STS.

The difference in the performance can be attributed to the biomechanics involved in the gait and balance components of the SPPB. These components are closely related to the quality of walking gait, as captured by the gait analyzer. In contrast, the 5x-STS, which heavily relies on hip flexor muscles, has relatively less influence on the results obtained from the gait analyzer.

#### FRAIL Versus Gait Analyzer

The FRAIL scale was categorized into functional and nonfunctional components. Only the FRAIL functional component was used in the gait analyzer diagnostic test. For the functional component versus the nonfunctional components, the results showed a higher specificity (0.45 vs 0.38) and TPR (0.45 vs 0.38). Both had the same PPV of 0.83; however, the FRAIL functional (0.64) and nonfunctional (0.68) AUC values were only fair (Table 2).

#### FROP-Com Versus Gait Analyzer

The FROP-Com test (Table 2) showed good specificity (0.91) and PPV (0.83) with an AUC of 0.68.

#### Gait Speed Versus Gait Analyzer

Gait speed (Table 2) showed good specificity (1.0) and PPV (1.0) with an AUC of 0.80.

#### TUG Versus Gait Analyzer

The TUG test (Table 2) showed very good sensitivity (1.0), specificity (0.94), TPR (1.0), and NPV (1.0) with an AUC of 0.97.

### Heart Rate Analyzer

#### SPPB Versus Heart Rate Analyzer

The SPPB (gait and balance) demonstrated excellent sensitivity, TPR, and NPV (all 1.00) with an AUC of 0.86.

#### FRAIL Versus Heart Rate Analyzer

The FRAIL (nonfunctional) reference standard showed excellent sensitivity, TPR, and NPV (all 1.00) with an AUC of 0.86.

#### FROP-Com Versus Heart Rate Analyzer

The FROP-Com test showed a fair AUC value of 0.75, and sensitivity, specificity, and TPR were 0.75. PPV and NPV were 0.60 and 0.86, respectively.

#### Gait Speed Versus Heart Rate Analyzer

Gait speed achieved a fair AUC value of 0.72 with specificity of 0.78 and NPV of 0.88.

#### Review of TUG Versus Heart Rate Analyzer

The TUG showed an AUC value of 0.86 with a high sensitivity, TPR, and NPV (all 1.0).

### Daily Living Analyzer

The daily activity levels (low, medium, or high) for each participant were calculated based on the energy level formula in the Methods section. The distribution of the daily activity level is shown in Table 3.

**Table 3:** Distribution of the 21 participants according to the daily living analyzer.

Classification	Distribution, n (%)
Low level	5 (23)
Medium level	4 (18)
High level	2 (59)

## Discussion

### Principal Findings

Our study showed that both the gait and heart rate analyzers were excellent predictors of functional domains and displayed high sensitivity for SPPB (gait and balance) and TUG. The gait analyzer also had superior performance for specificity in the same domains. The heart rate analyzer was a good predictor of the FRAIL nonfunctional domains, whereas the gait analyzer was a good predictor of the functional domains except for the 5x-STS. Compared with more traditional methods of performing multiple functional tests and frailty screening, wearables may be more efficient and cost effective but require validation at the population level.

### Interpretation of Findings

Widely adopted assessment tests like the SPPB, FRAIL, FROP-Com, gait speed, and TUG provide a good platform to assess the risk of falls and frailty among older adults but require trained human resources, equipment, and space and are often conducted in a controlled setting that may not accurately reflect day-to-day activities. Advances in wearable technologies provide longitudinal and continuous monitoring of daily living activities in participants’ natural living environments and may provide a better quantification of exhaustion, slowness, and weakness [29]. Prior studies that validated the use of sensors for measuring 5x-STS either used multiple sensors or incorporated a machine learning algorithm with a 2-sensor configuration for detecting frailty [29,30]. Wearable devices offer superior data collection in terms of continuity and reliability. They can operate remotely and autonomously and are a very cost-effective, efficient tool.

In this study, 43% of the participants fell into the low-to-medium activity level category. This is comparable with other studies where about 35% of community-dwelling older adults self-reported low activity levels [31]. Evaluation of gait quality using triaxial accelerometers on the lower back in addition to daily activities have been shown to improve the prediction of future falls [32]. Similar to low heart rate variability, low HRR is a recognized biomarker for frailty, as it is associated with chronic diseases, physical function, lower cardiovascular fitness reserve, chronic inflammation, and mortality [16,24,33]. Unintentional weight loss in frailty could be due to underlying inflammation, which could be the underlying cause for lower HRR. Qiu et al [16] showed that, for every 10-bpm HRR decrease, the risk of cardiovascular events increases by 13% and the risk of all-cause mortality increases by 9%, further supporting our study findings on the validation of HRR to screen for nonfunctional components of frailty.

Falls are the second leading cause of death due to injury after road traffic accidents and a major public health problem [34-36]. In Canada and the United States, up to one-third of older adults ≥65 years old fall each year, one-half of whom may experience recurrent falls [37]. There are many fall risk assessment tools such as the Activity-specific Balance Confidence Scale, FROP-Com, and TUG, but most of them are either questionnaire-based or provide measurements at a specific time point (eg, in a clinic or hospital) [38]. Questionnaire-based falls risk assessments have lower predictive value [14]. The FROP-Com only has a moderate capacity to predict falls and, when validated against the TUG, has an AUC of 0.63 (95% CI 0.57-0.69) [25]. Predictability can be improved by combining it with functional-based measures. The TUG, although recommended by the US Centers for Disease Control and Prevention to screen for falls risk, is recognized mainly as a measure of balance and is useful for ruling in rather than ruling out falls in high-risk individuals [39,40]. Asai et al [41] showed that the combination of dual tasking and TUG measurements was associated with falls in the old-old but not the young-old. Sensor-based falls risk assessment has gained significant traction in recent years, as it measures data collected in a normal life setting [39]. However, most of the data obtained through sensors are validated against a falls risk classification or fall history. Identification of fallers using sensor-based assessments is dependent on the activity or task and the location of the sensors. Sensors are worn in various locations such as the sternum, waist, and head, which may not be acceptable to most people and may result in gender-specific differences [19]. Most published studies that have validated sensor-based falls risk assessments using gait speed, 5x-STS, or TUG have been conducted in an experimental setting or measured only gait speed in real life [29,32]. Activities such as walking and turning around or dual-task walking have higher predictive value than walking in a straight path [41,42]. Multilocation sensors performed better than those in a single location [14]. Although our study is one of the first to validate the gait and heart rate analyzers with FROP-Com using a single sensor on the ankle, the wearable was not able to differentiate the walking terrain, and we had no longitudinal data to show if a longitudinal assessment using a wearable was superior at predicting falls than time-point assessments in the clinic setting.

### Limitations and Future Direction

Our study is one of the first few to show correlations between the gait and heart rate analyzers and frailty, gait speed, SPPB, TUG, and falls risk. However, there are several limitations that warrant mentioning. Walking gait data were collected through daily living activities, and a high variation of walking patterns was found. To standardize the walking pattern for this study, only steps that were part of a continuous 40 steps or more were tagged by the gait analyzer. Since the wearable also allows continuous tracking of walking, this analyzer is capable of aggregating a much larger amount of gait characteristic data than the specific time point data collected using gait speed or the TUG test. Second, the gait analyzer was used with a small sample of individuals who had fallen or were at a high risk of falls. The findings need to be validated at the larger population level. Third, the wearable we used needs to be worn on the ankle, which may be inconvenient for older adults, and is not completely waterproof. Fourth, we had no measures of muscle strength as a proxy for weakness for the diagnosis of frailty. In addition, we had no longitudinal outcome data for frailty; the data collected are only meaningful for moderate and high activity levels. For low activity levels, the activities were not well understood. In addition, for walking activities, we could not differentiate between walking on stairs and over uneven terrains. Having additional user log capabilities (for participants with low activity levels) and better detection of the walking environment (stairs or uneven terrain) could provide additional insights into daily activities. Last, none of the reference tools are considered the gold standard for frailty or falls evaluation. Without longitudinal outcome data, we cannot correlate our wearable data with long-term outcomes.

Our study showed that agreement between the gait and heart rate analyzers and the functional component of the FRAIL scale, gait speed, and FROP-Com was significant. In addition, the heart rate analyzer had significant agreement with the nonfunctional component of the FRAIL scale. As shown in prior studies, measuring heart rate dynamics or variation, in conjunction with physical activity, can be a good indicator of frailty [20,22]. It would be interesting to explore machine learning protocols incorporating the different parameters and HRR to improve the prediction of frailty and falls as well as a long-term follow-up to determine the associations with long-term outcomes such as disability, falls, and mortality. In addition, the next-generation design will need to be user-friendly with a better design and an algorithm to detect those with low activity levels and the walking terrain. Another aspect of wearables that will need to be evaluated longitudinally is their role in behavior change toward a healthier lifestyle, through triggers, goal setting, and prompts toward achieving sustainable goals [43].

### Conclusion

Our study showed significant correlations between the gait and heart rate analyzers and physical performance, frailty, and falls risk in older adults at risk of falls. Next-generation wearables will need to be validated at the population level; incorporate a better design; be able to detect walking terrains; and integrate the gait, heart rate, and daily living analyzers with immediate results.

## Appendix

Multimedia Appendix 1 Workflow, diagnostic test, cutoff thresholds, and area under the curve (AUC) calculation.

## Abbreviations

AUC: area under the curve
FROP-Com: Falls Risk for Older People in the Community
HRR: heart rate recovery
NPV: negative predictive value
PCB: printed circuit board
PPV: positive predictive value
ROC: receiver operating characteristic
SPPB: Short Physical Performance Battery
STS: sit-to-stand
TPR: true positive rate
WHO: World Health Organization

## Data Availability

The data sets used in the current study are available from the corresponding author on reasonable request.
