# Supplementary material for: Ability of Heart Rate Recovery and Gait Kinetics in a Single Wearable to Predict Frailty: Quasiexperimental Pilot Study
Source: JMIR Form Res. 2024 Oct 3;8:e58110. doi: 10.2196/58110 (PMC11487206; doi:10.2196/58110)
Supplement: Multimedia Appendix 1 [file formative_v8i1e58110_app1.docx]

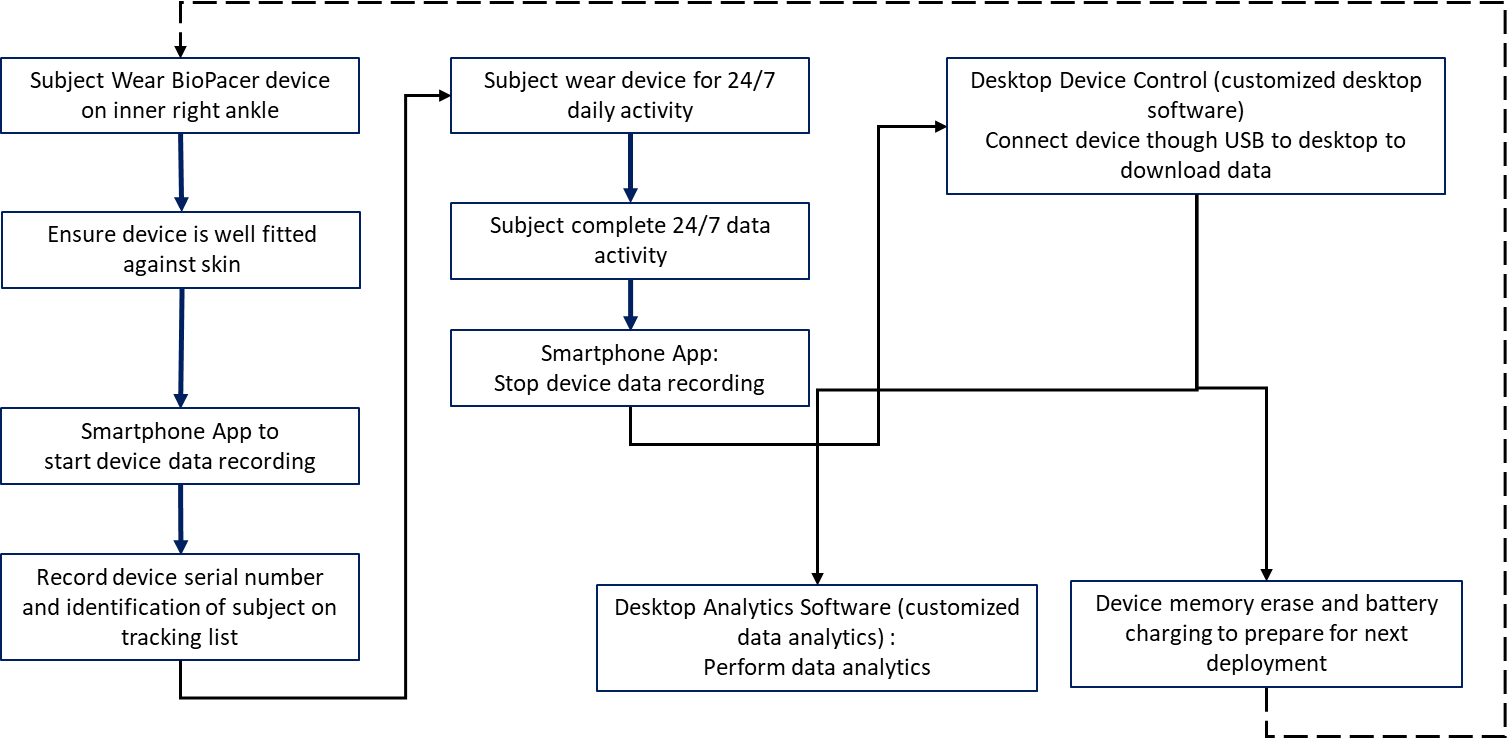


**Figure S1.** Workflow of wearable device for deployment, retrieval, and post-data analysis.


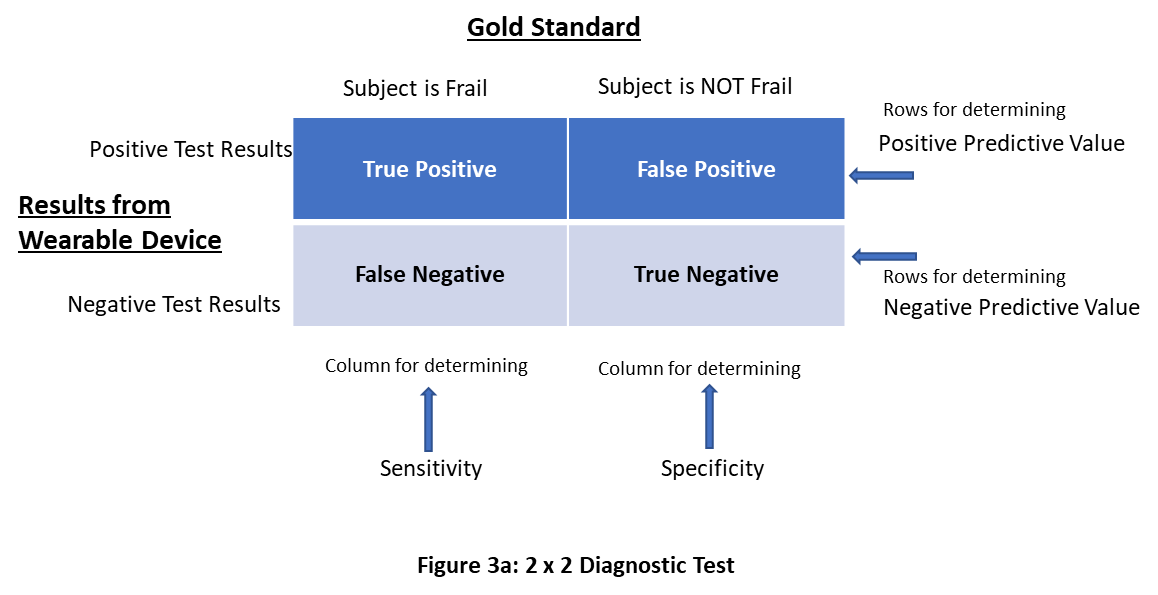


**Figure S2.** 2 × 2 Diagnostic Test


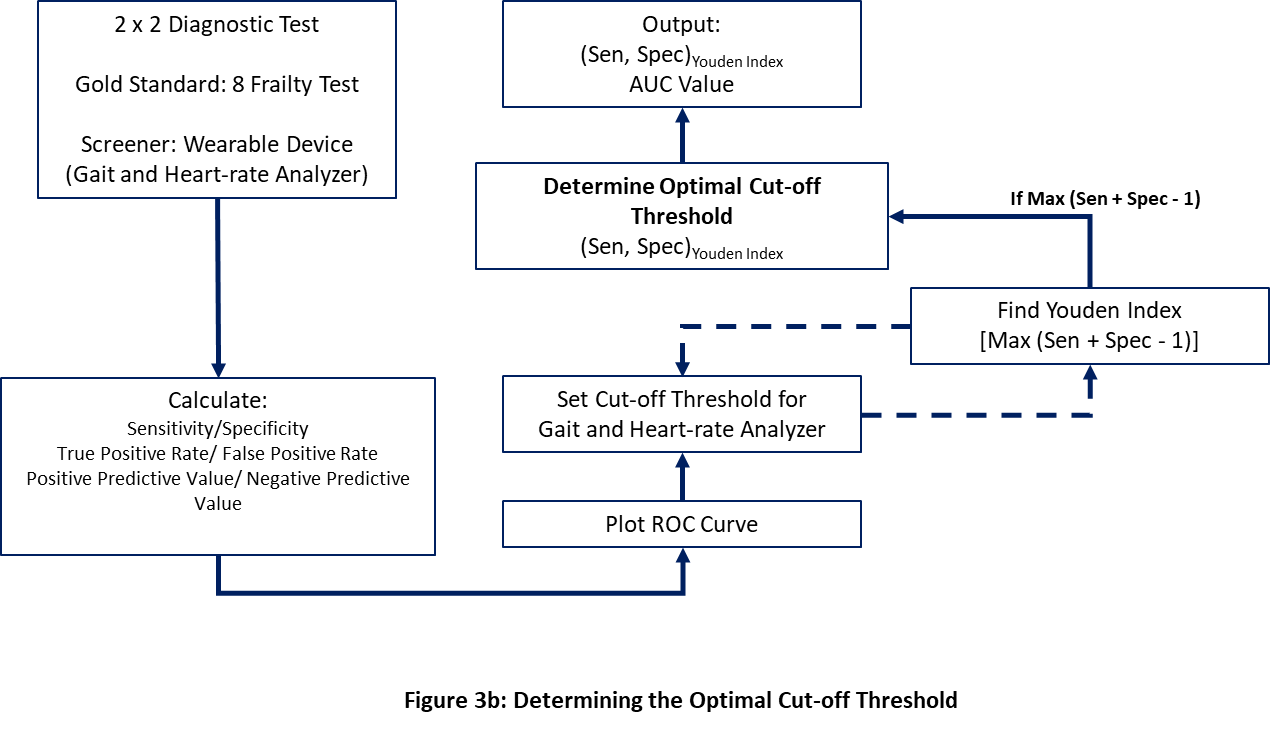


**Figure S3.** Determining the Optimal Cutoff Threshold


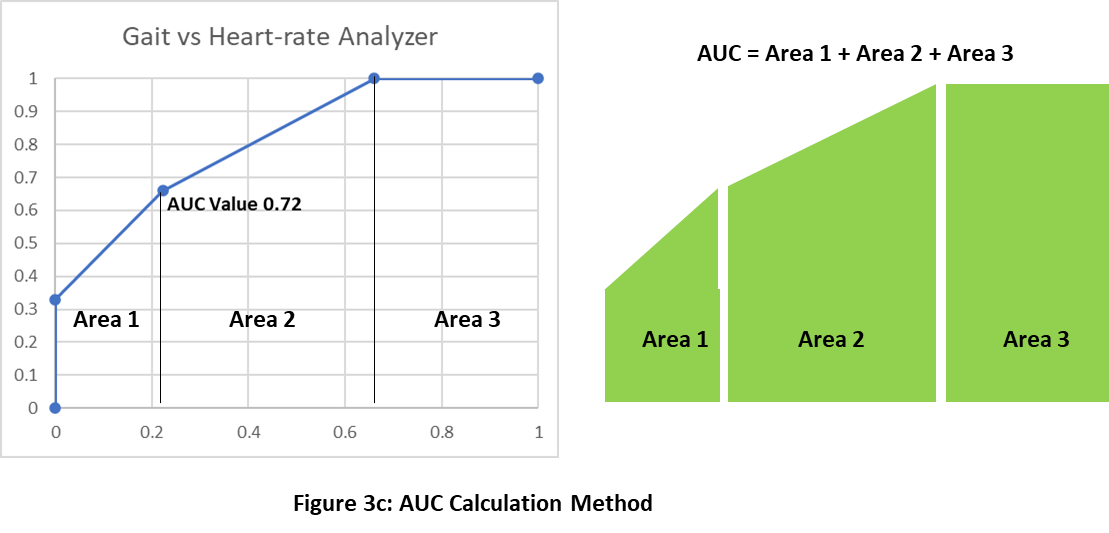


*Area of Trapezoid = (a + b) x h* $\div$ *2 (a and b = base, and h = height)*

**Figure S4.** AUC Calculation Method
